# Decreased Expression of PACSIN1 in Brain Glioma Samples Predicts Poor Prognosis

**DOI:** 10.3389/fmolb.2021.696072

**Published:** 2021-08-05

**Authors:** Zhou Zimu, Zhang Jia, Fu Xian, Ma Rui, Ren Yuting, Wei Yuan, Wen Tianhong, Ma Mian, Liu Yinlong, Shan Enfang

**Affiliations:** ^1^ School of Nursing, Nanjing Medical University, Nanjing, China; ^2^ Cancer Nursing Research Branch, Nursing Research Center, Nanjing Medical University, Nanjing, China; ^3^ Department of Neurosurgery, The Affiliated Suzhou Hospital of Nanjing Medical University, Suzhou Municipal Hospital, Gusu School, Nanjing Medical University, Suzhou, China; ^4^ Department of Neurosurgery, The Affiliated Huashan Hospital, Fudan University, Shanghai, China

**Keywords:** PACSIN1, glioma, os, biomarker, targeted therapy

## Abstract

Gliomas are the most severe brain tumours with a poor prognosis. Although surgery, postoperative radiotherapy and chemotherapy can improve the survival rate of glioma patients, the prognosis of most glioma patients is still poor. In recent years, the influence of gene-targeted therapy on gliomas has been gradually discovered, and intervening the occurrence and development of brain gliomas from the perspective of the gene will significantly improve treatment prognosis. Protein Kinase C and Casein Kinase Substrate in Neurons 1 (PACSIN1) is a member of the conserved peripheral membrane protein family in eukaryotes. Improper expression of PACSIN1 can lead to neurological diseases such as Huntington’s disease and schizophrenia. However, its relationship with tumours or even gliomas has not been explored. The study aims to explore PACSIN1 as a prognostic factor that can predict overall survival (OS) for gliomas. We collected the data from CGGA, TCGA, GEO databases and the pathological glioma tissue specimens from 15 clinical glioma patients surgically resected. The differential expression of PACSIN1 in various clinical indicators, the genes related to PACSIN1 expression, the prognostic value of PACSIN1 and the functional annotations and pathway analysis of differently expressed genes (DEGs) were analysed. The results revealed that PACSIN1 had low expression levels in grade IV, IDH1 wild-type and 1p/19q non-codel group gliomas, and PACSIN1 was considered a mesenchymal molecular subtype marker. PACSIN1 expression is positively correlated with OS in all gliomas and it was found that PACSIN1 influenced the occurrence and development of gliomas through synaptic transmission. The PACSIN1 expression is negatively correlated with the malignant degree of gliomas and positively associated with the OS, indicating that PACSIN1 would play an essential role in the occurrence and development of gliomas and might be a potential new biomarker and targeted therapy site for gliomas.

## Introduction

Glioma is a primary tumour developed by glial cells of the brain or central nervous system, accounting for 24% of the total number of primary brain and central nervous system tumours (Davis, 2018). Among them, Glioblastoma (GBM) is the most common primary malignant tumour of the central nervous system, with poor prognosis. Due to its highly malignant biological behaviours such as high proliferation and invasiveness, it severely limits the overall survival (OS) of tumour patients (Brat et al., 2015; Schalper et al., 2019).

High grades, Isocitrate dehydrogenase 1 (IDH1) wide-type, 1p/19q non-codel gliomas represent poor prognosis (Louis et al., 2014; Louis et al., 2016; Hu et al., 2017; Chang et al., 2018). At present, the treatment of gliomas is mainly surgical resection, plus radiotherapy, chemotherapy and other treatment methods. However, the effect is still not ideal, and the survival time of patients still cannot be effectively extended (Lim et al., 2018). It has been shown that gene-targeted therapy has an important impact on the survival of patients for many cancers (Hyman et al., 2015; Long et al., 2016). Moreover, many studies have shown that targeted therapy can improve the therapeutic effect of gliomas (Lin et al., 2017). Intervention in the occurrence and development of brain gliomas from the perspective of the gene will significantly improve the effectiveness of treatment. The pathogenesis and prognosis of gliomas are related to many factors. Exploring new biomarkers of gliomas helps predict biological behaviour, and the new biomarkers help patients design personalized treatment projects and develop new therapeutic targets.

Protein Kinase C and Casein Kinase Substrate in Neurons 1 (PACSIN1), also known as SYNDAPIN1, is a member of the PACSIN family. Studies have demonstrated that the PACSIN protein family is a conservative peripheral membrane protein family in eukaryotes and plays an essential role in the synaptic vesicle transport cycle and receptor-mediated endocytosis (Anggono et al., 2006; Clayton et al., 2009). Meanwhile, it can bind to tubulin to promote the assembly of microtubules (Grimm-Günter et al., 2008), and also plays a particular role in membrane shaping and reconstruction, which is crucial for the formation of neural morphology (Quan and Robinson, 2013). Besides, PACSIN1 plays roles in regulating interferon response (Schwintzer et al., 2011; Esashi et al., 2012). It can even inhibit synapses, regulate axonal elongation and branch, so as to regulate nerve development and nervous system disorders (Liu et al., 2012; Mahadevan et al., 2017). However, its role and function in tumours are still unclear. So far, many studies have found that PACSIN1 plays an essential role in developing of neurons and the regulation of the nervous system (Pérez-Otaño et al., 2006), and the inappropriate expression of PACSIN1 can even lead to Huntington’s disease, schizophrenia and other neurological diseases (Gopalakrishnan et al., 2016; Koch et al., 2020). Therefore, we hypothesized that PACSIN1 expression is related to the occurrence and development of gliomas.

Our study collected and extracted data of clinical indicators and gene expression of gliomas from Chinese Glioma Genome Atlas (CGGA), The Cancer Genome Atlas (TCGA), Gene Expression Omnibus (GEO) databases and clinical samples, gradually analyzed the influence of PACSIN1 expression on the related clinical indicators of gliomas, studied its clinical significance and explored the mechanism of PACSIN1 affecting gliomas, so as to provide the experimental basis for the diagnosis, prognosis, development and treatment of gliomas.

## Materials and Methods

### General Information

The clinical and gene expression data were downloaded from the mRNAseq_325 and mRNA sequencing samples (non-glioma as control) in CGGA (http://www.cgga.org.cn/). And we also downloaded 702 clinical and gene expression data of gliomas from TCGA (https://portal.gdc.cancer.gov/) and the data of Platform and Series matrix of 684 patients in three datasets (GSE4290, GSE16011 and GSE58218) of glioma samples from GEO (https://www.ncbi.nlm.nih.gov/geo/). The RStudio (version4.0.1) and Perl software (version5.30.2.1) were used to collate CGGA, TCGA and GEO datasets, including annotation, merge, the complement of missing values, background correction, and data standardization of raw data.

### Data Set Filtering

When conducting clinical analysis, the CGGA dataset’s data included grades, IDH1 mutation status, 1p/19q codeletion status, different molecular subtypes defined by TCGA network (Verhaak et al., 2010), survival status, survival time and PACSIN1 expression. The data collected from the TCGA dataset included grades, IDH1 mutation status, 1p/19q codeletion status, survival status, survival time and PACSIN1 expression. The data collected from the GEO datasets included grades, IDH1 mutation status, 1p/19q codeletion status and PACSIN1 expression.

### Correlation Analysis

Pearson correlation analysis was performed to retrieve the genes significantly associated to the PACSIN1 expression, and the heatmap package of R language was used to list the differently expressed genes (DEGs). Gene Ontology (GO) and the Kyoto Encyclopedia of Genes and Genomes (KEGG) performed the functional annotations of the DEGs and analyzed gene signaling pathways using clusterProfiler, org. Hs.eg.db and pathview packages of R language. Then, PACSIN1 and DEGs were uploaded to the STRING website (https://string-db.org/) to build a protein-protein interaction (PPI) network (Szklarczyk et al., 2017). The analysis results were imported into Cytoscape v3.8.0 software and cluster analysis was carried out by Molecular Complex Detection (MCODE) plugin to find out the major gene clusters in the network (Bader and Hogue, 2003), and then the genes with high scores were further analyzed to explore their related functional annotations and signaling pathways.

### Clinical Sample Collection

Total 15 patients with glioma (5 cases in each grade-Ⅱ Ⅲ Ⅳ) were randomly selected from The Affiliated Suzhou Hospital of Nanjing Medical University. We took the specimen of the patient’s surgically resected pathological glioma tissues. Inclusion criteria: ①All patients were diagnosed as glioma by definite examination, which followed surgical treatment indications; ②All patients underwent a single glioma resection. ③All patients volunteered to participate in this study and signed the informed consents. Exclusion criteria: ①Patients with a history of the tumour; ② Patients with concurrent multiple tumours; ③Patients with other operations during glioma resection. ④Patients whose postoperative pathology cannot make an exact diagnosis. This study was approved by the Ethics Committee of Nanjing Medical University**.**

### Immunohistochemical (IHC)

The tumor sample of patients with glioma were made into paraffin sections, dewaxed with xylene, rehydrated with gradient alcohol, and boiled in the citrate antigen solution. After washed with PBS, the sections were incubated with 10% sheep serum and PACSIN1 antibody (Proteintech Cat# 13219-1-AP, RRID: AB_10637851) at 4°C overnight. After that, the sections were incubated with HRP-conjugated secondary antibody (Proteintech Cat# SA00001-2, RRID: AB_2722564), set at room temperature for 1 h, incubated with DAB (BOSTER Cat# AR1000) for 10 min, soaked in hematoxylin for 3 min, washed with PBS, and then sealed. The staining results were put under a microscope (BX53, Olympus, Tokyo, Japan) to find the expression differences of cancer tissues. At least three tumour sections were analysed for each patient.

### Statistical Methods

Kruskal-Wallis rank sum test was performed to detect the expression difference of PACSIN1 among patients with different glioma grades and different molecular subtypes defined by the TCGA network (Verhaak et al., 2010). Wilcoxon rank-sum test was performed to detect the expression difference of PACSIN1 among patients with different IDH1 mutation status and 1p/19q codeletion status. Kaplan-Meier analysis was performed to investigate the prognostic value of PACSIN1. Moreover, Cox proportional hazards model analysis was used to verify PACSIN1 as an independent prognostic factor and R language packages (GGplot2, pROC, PheatMap, and Corrgram) was applied for other statistical calculations and graph drawing. All differences were considered statistically significant at the level of *p* < 0.05.

## Results

### Protein Kinase C and Casein Kinase Substrate in Neurons 1 Significantly Downregulates in the Tumor Group, and Decreases With the Progression of Glioma Grades

**FIGURE 1:**
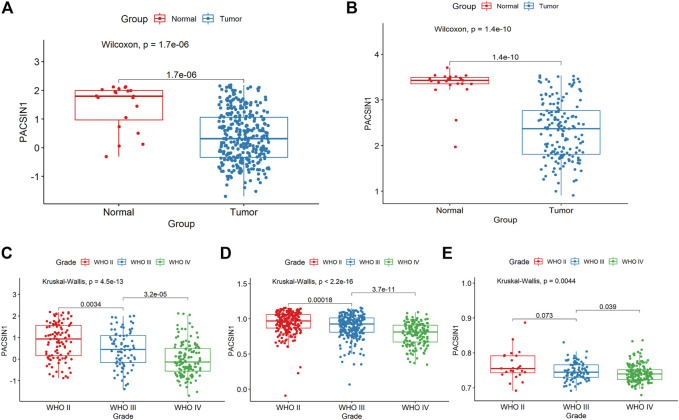
PACSIN1 expression in glioma group and normal group and in different grades. PACSIN1 expression was lower in tumor group and higher in normal group in CGGA **(A)** and GEO4290 datasets **(B)**. PACSIN1 expression decreased with the increase of grades in CGGA **(C)**, TCGA **(D)** and GSE 16011 datasets **(E)**.

In order to search for targeted molecular markers related to brain gliomas and evaluate their potential as molecular markers, we collected gene data of 325 tumour tissue samples and 20 normal tissue samples in CGGA and 157 tumour tissue samples and 23 normal tissue samples in GSE4290 dataset of GEO in the early stage. Softwares such as Rstudio and Perl were used to annotate the data, supplement the missing values, correct the background, and standardize the data. We detected and analyzed the DEGs in the tumour group and the normal group from the total gene expression profile perspective. Among these DEGs, PACSIN1, one member of the conservative peripheral membrane brain protein family in eukaryotes, caught our attention. The expression of PACSIN1 is lower in gliomas compared with normal group by analyzing CGGA and GSE4290 datasets (Figures 1A,B).

**FIGURE 2:**
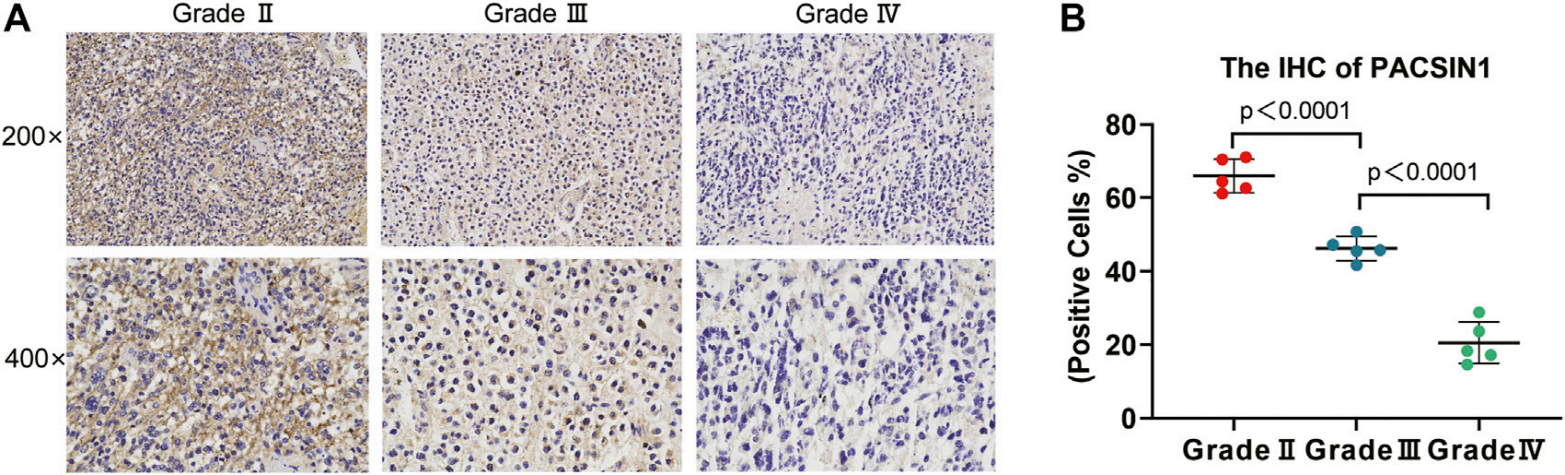
PACSIN1 IHC results in different grades. PACSIN1 protein expression staining results (semi-quantitative scoring, expression intensity × expression area): upper, ×200; lower, ×400 **(A)** suggested that PACSIN1 protein expression downregulated with the progression of grades **(B)**. Blue purple represents the nucleus stained with hematoxylin and brown yellow represents the expression of PACSIN1.

The PACSIN1 expression in glioma grades was extracted from CGGA mRNAseq_325 samples. After data collation, Kruskal-Wallis rank sum test was conducted to detect the expression difference of PACSIN1 among patients with different glioma grades. The results showed that the mRNA expression of PACSIN1 was negatively correlated with glioma grades (*p* < 0.0001) and was significantly lower in the grade IV group (Figure 1C). The results were verified in the TCGA dataset and 276 samples from the GSE16011 dataset (Figures 1D,E). In order to further verify the correlation between PACSIN1 and glioma grades, we collected pathological tissue samples from 15 patients in different grades. IHC results was consistent with database results that PACSIN1 protein expression was negatively correlated with glioma grades and there was statistical significance among each grade (*p* < 0.0001). The higher the glioma grade, the lower the PACSIN1 protein expression (Figure 2). The PACSIN1 protein expression in the grade IV group was significantly lower than that in the grade Ⅱ and Ⅲ group (*p* < 0.0001). The results indicated that PACSIN1 plays a vital role in the progression of gliomas.

### Protein Kinase C and Casein Kinase Substrate in Neurons 1 Raises in IDH1-Mutant Gliomas, and Reduces in 1P/19q Non-codel Gliomas

**FIGURE 3:**
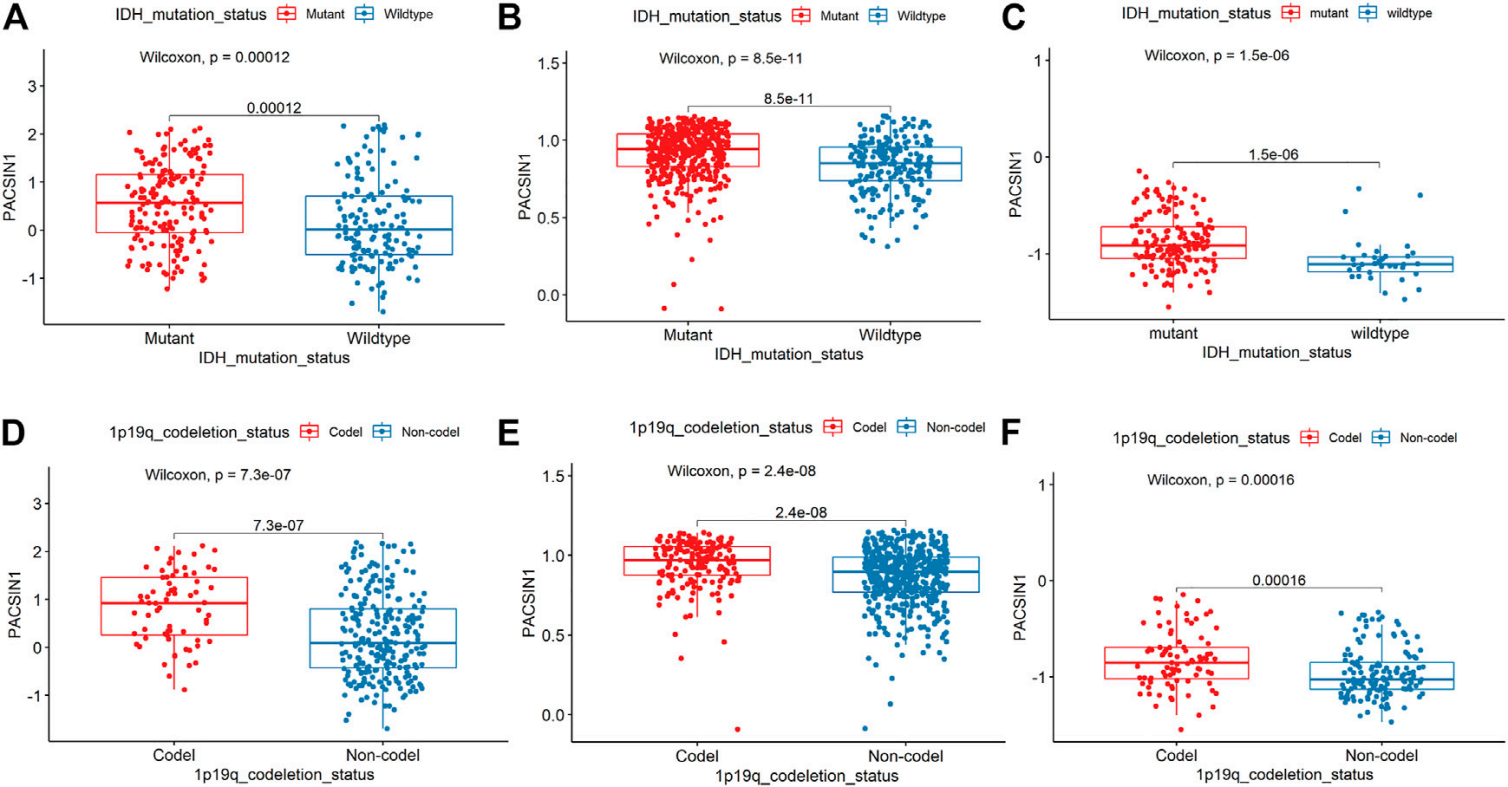
PACSIN1 expression in IDH1 mutation status and 1p/19q codeletion status. PACSIN1 upregulated in the IDH1-mutant group in CGGA **(A)**, TCGA **(B)**, GSE 58218 datasets **(C)** and downregulated in non-codel group in CGGA **(D)**, TCGA **(E)**, GSE 58218 datasets **(F)**.

The PACSIN1 expression in IDH1 mutation status and 1p/19q codeletion status was extracted from the CGGA mRNA_325 sample. IDH-mutant gliomas have a better prognosis than IDH wild-type gliomas (Chang et al., 2018). The results indicated that PACSIN1 expression in IDH1-mutant group was significantly higher than that in IDH1 wild-type group (Figure 3A, *p* < 0.001). Compared with the same pathological type of patients without defect, patients with 1p/19q codel have higher sensitivity to chemotherapy and longer survival time (Hu et al., 2017). As shown in Figure 3D, the PACSIN1 expression in 1P/19q non-codel group was significantly lower than that in the 1p/19q codel group (*p* < 0.0001). The results were verified in TCGA and 228 samples from GSE58218 dataset (Figure 3B,C,E,F). All the above results suggested that PACSIN1 upregulated in IDH1-mutant gliomas and downregulated in 1P/19q non-codel gliomas.

### Protein Kinase C and Casein Kinase Substrate in Neurons 1 Decreases in Mesenchymal Molecular Subtype Gliomas and can be Considered as a Potential Biomarker

**FIGURE 4:**
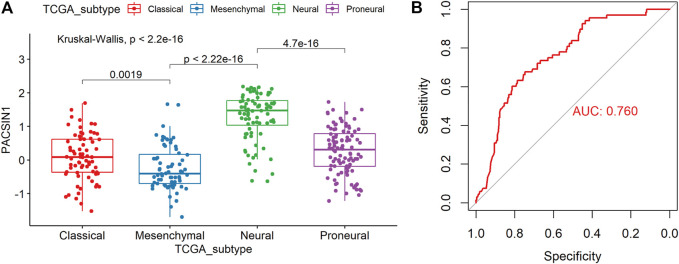
PACSIN1 expression in different molecular subtypes defined by TCGA network. PACSIN1 was decreased in mesenchymal molecular subtype in CGGA dataset **(A)**. ROC curve of PACSIN1 expression to predict mesenchymal molecular subtype in CGGA dataset **(B)**.

In order to explore the PACSIN1 expression in gliomas further, we analyzed the PACSIN1 expression in different molecular subtypes (Classical, Mesenchymal, Neural, and Proneural) defined by the TCGA network (Verhaak et al., 2010). The results revealed that the PACSIN1 expression in mesenchymal molecular subtype decreased significantly more than other subtypes, and there were significant differences between these subtypes (*p* < 0.0001, Figure 4A). To further verify this finding, we performed the receiver operating characteristic curve (ROC) to verify the relationship between PACSIN1 expression and gliomas at all grades of the mesenchymal molecular subtype. In the CGGA dataset, the area under the curve (AUC) was 76.0%, indicating that PACSIN1 has a particular predictive ability (Figure 4B).

### Protein Kinase C and Casein Kinase Substrate in Neurons 1 Is Proportional to the OS and Could Be Used as an Independent Prognostic Factor for the OS of Glioma Patients

**FIGURE 5:**
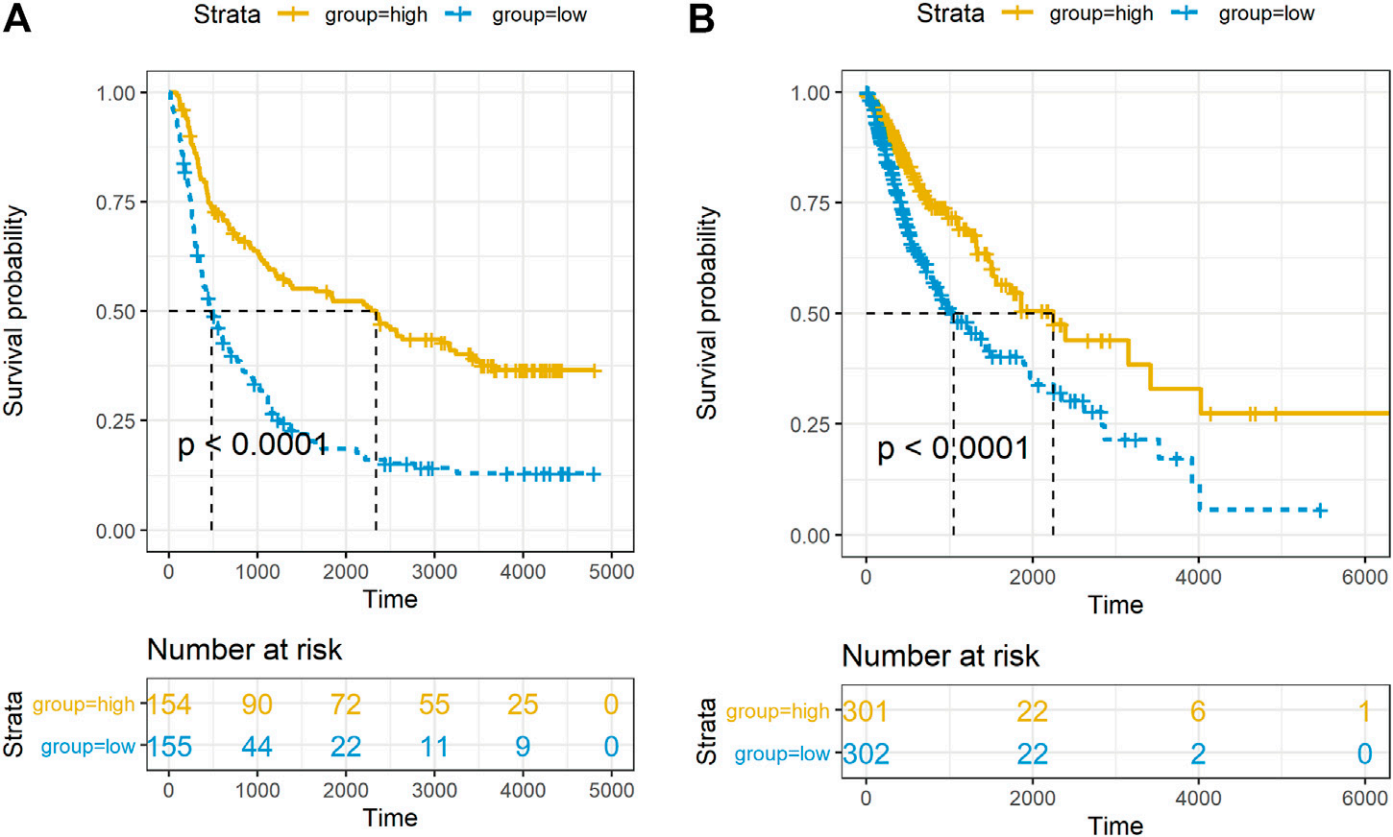
Relationship between PACSIN1 expression and survival status and time. Kaplan-Meier estimates of survival for all grades in the CGGA **(A)** and TCGA datasets **(B)**.

In order to explore the impact of PACSIN1 on the survival of glioma patients, we analyzed the PACSIN1 expression in the survival status and survival time of 325 samples from the CGGA dataset and 603 samples from the TCGA dataset. When observing the survival time of each group in the CGGA dataset, the results explained that the patients with low expression of PACSIN1 significantly shortened OS in all grades of gliomas (Figure 5A, *p* < 0.0001). Similar results were obtained in the TCGA dataset (Figure 5B, *p* < 0.0001). Then, to determine whether PACSIN1 expression could act as an independent prognostic factor, univariate and multivariate Cox regression analyses of CGGA dataset were performed (Table 1). Univariate regression analysis results showed that PACSIN1 (*p* < 0.0001), with all clinical indicators including age (*p* < 0.0001), IDH1 mutation status (*p* < 0.0001), 1P/19q codeletion status (*p* < 0.0001), and grade (*p* < 0.0001) could be used as a predictor of OS of gliomas at all grades. Hazard ratio (HR) is an expression of the hazard or chance of events occurring in the treatment arm as a ratio of the hazard of the events occurring in the control arm. Hazard ratios are commonly used in survival analysis to allow hypothesis testing (Blagoev et al., 2012; Sashegyi and Ferry, 2017). In Table 1, hazard ratio of PACSIN1<1. This indicates that the higher the gene expression, the longer the survival time of patients.

**TABLE 1:** Univariate and multivariate Cox analysis of PACSIN1 expression and clinical indicators in CGGA dataset.

Clinical factors	Univariate			*p*	Multivariate			*p*
	HR	95%CI			HR	95%CI		
		Lower	Upper			Lower	Upper	
Age	1.033	1.020	1.046	<0.0001	1.013	1.000	1.025	0.047
Gender (Male)	0.935	0.709	1.232	0.631	—	—	—	—
IDH mutation status (Mutant)	0.355	0.268	0.470	<0.0001	1.013	0.719	1.429	0.940
1p/19q status (Non-codel)	5.887	3.608	9.606	<0.0001	3.803	2.254	6.417	<0.0001
Grade (WHO IV)	4.758	3.536	6.402	<0.0001	2.734	1.942	3.848	<0.0001
High PACSIN1 expression	0.555	0.475	0.649	<0.0001	0.741	0.633	0.868	<0.0001

In the results of multivariate regression analysis, PACSIN1 showed significant results (*p* < 0.0001) in the evaluation of age (*p* = 0.047), IDH1 mutation status (*p* = 0.940), 1P/19q codeletion status (*p* < 0.0001), and grade (*p* < 0.0001). The results suggested that PACSIN1 could be used as an independent prognostic factor.

### Protein Kinase C and Casein Kinase Substrate in Neurons 1 Affects the Occurrence and Development of Gliomas by Interfering With Synaptic Transmission

**FIGURE 6:**
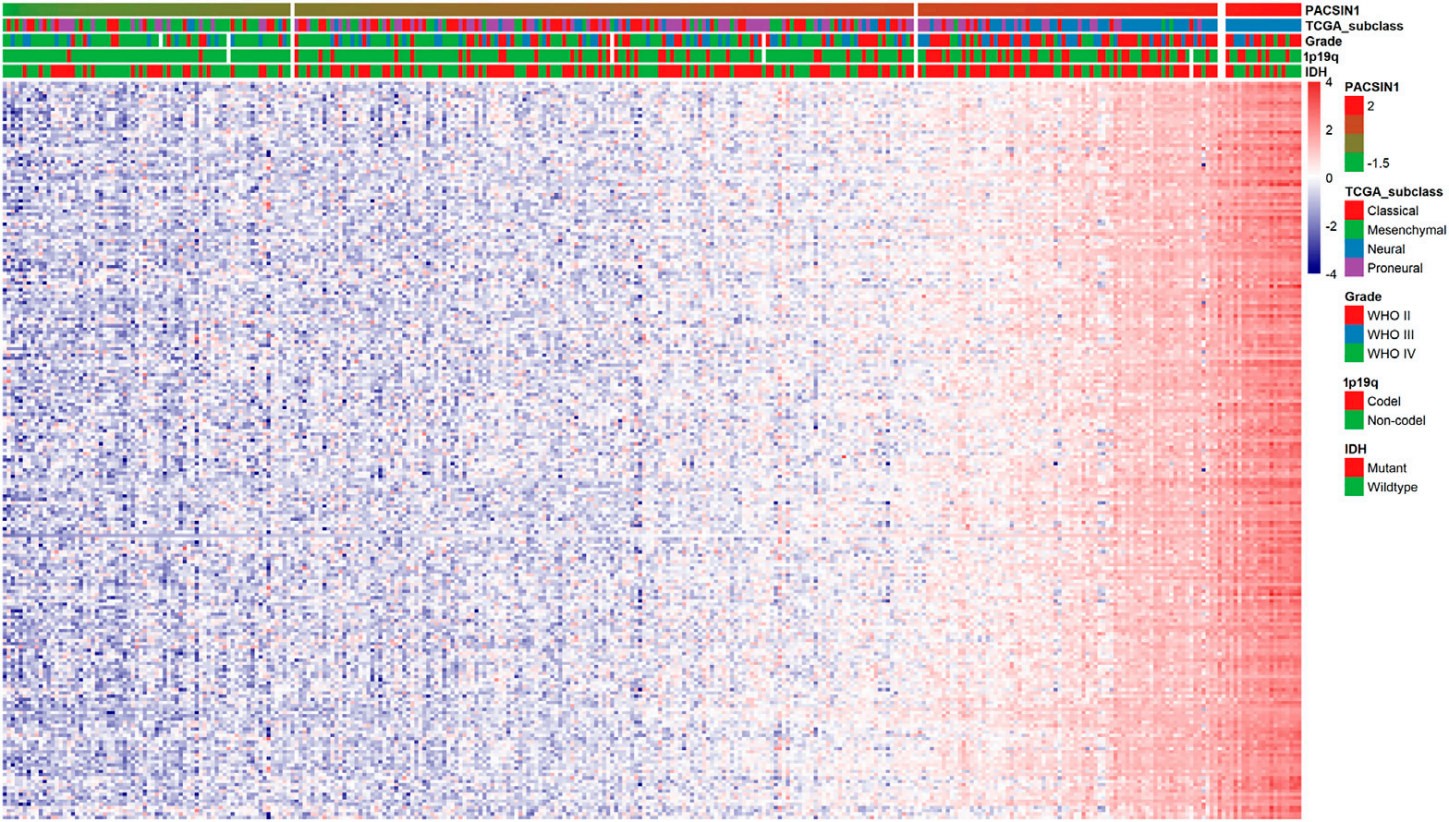
Correlations of 224 DEGs expression with the clinical indicators in gliomas in CGGA dataset.

**FIGURE 7:**
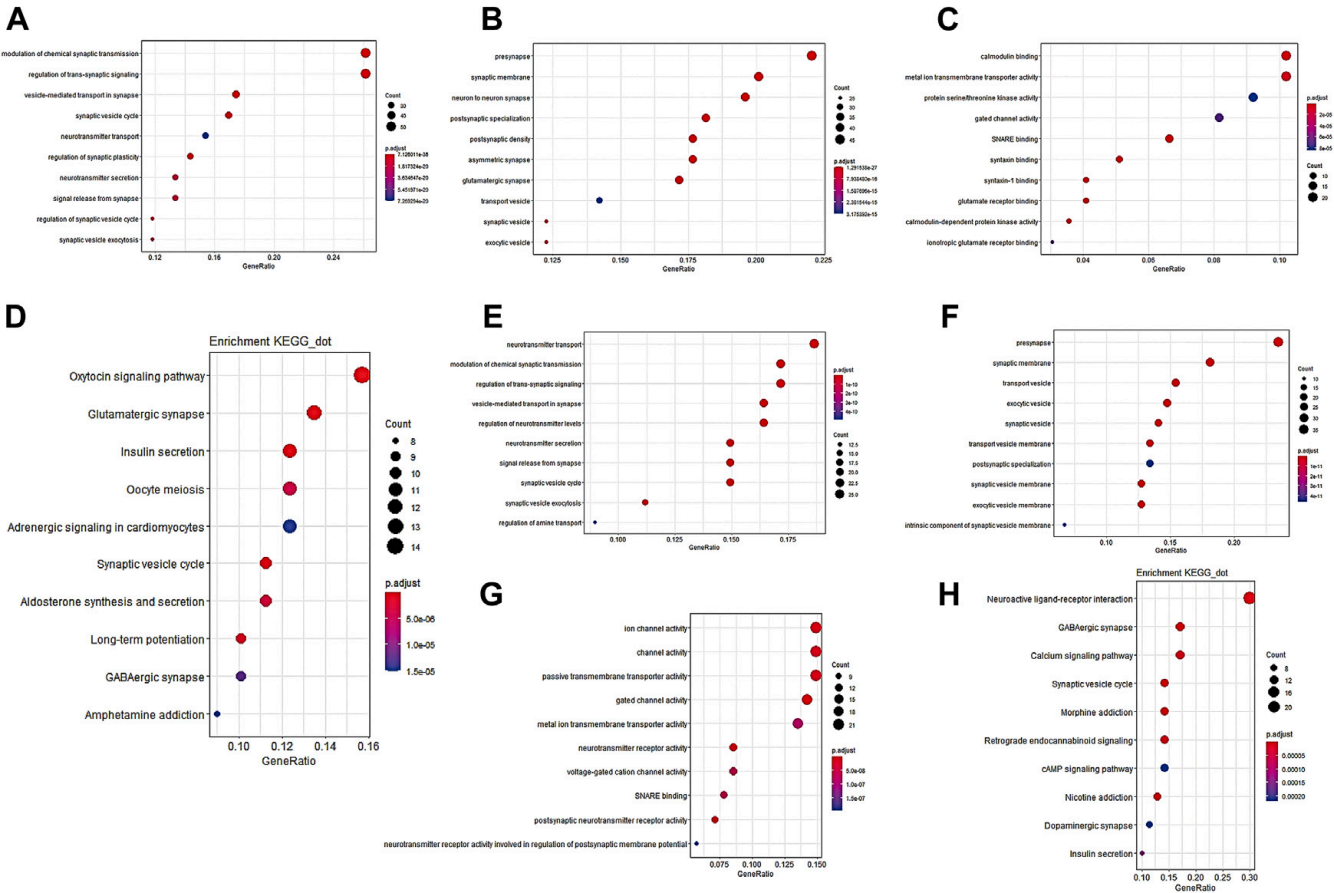
GO function annotation and KEGG pathway analysis results of 224 DEGs. DEGs in CGGA dataset GO pathway enrichment analysis results BP **(A)**, CC **(B)**, MF **(C)**, KEGG pathway enrichment analysis results **(D)**. DEGs in TCGA dataset GO pathway enrichment analysis results BP **(E)**, CC **(F)**, MF **(G)**, KEGG pathway enrichment analysis results **(H)** P. adjust: *p* value of gene enrichment. Counts: number of genes in a cluster of DEGs that belong to this pathway.

Finally, we preliminarily explored the possible mechanism by which PACSIN1 inhibits the progression of gliomas. First, Pearson correlation analysis was performed between the expression of PACSIN1 and other genes in the genomic map of the CGGA dataset in 325 patients. The results showed that 224 genes (R > 0.8) were significantly correlated with the PACSIN1 expression (Figure 6). Next, GO function annotation and KEGG pathway analysis were performed on the DEGs. GO enrichment analysis was performed on the biological process (BP), cellular component (CC) and molecular function (MF) (Figures 7A–C, B and C). Based on GO three groups’ analysis results, the DEGs were mainly enriched in the modulation of chemical synaptic transmission, regulation of trans-synaptic signaling, presynapse, synaptic membrane and neuron to neuron synapse. KEGG pathway analysis results showed that the DEGs were mainly enriched in Glutamatergic synapse, Calcium signaling pathway, Insulin secretion, Synaptic vesicle cycle and GABAergic synapse and other pathways (Figure 7D). In addition, we also performed a Pearson correlation analysis on the PACSIN1 expression and other genes in the whole genome map of the TCGA dataset, and 167 DEGs were obtained (R > 0.8). Meanwhile, GO function annotation and KEGG pathway analysis were also performed, and the results were shown in Figures 7E–H. The results indicated that PACSIN1 may regulate synaptic transmission to affect the occurrence and development of gliomas.

**FIGURE 8:**
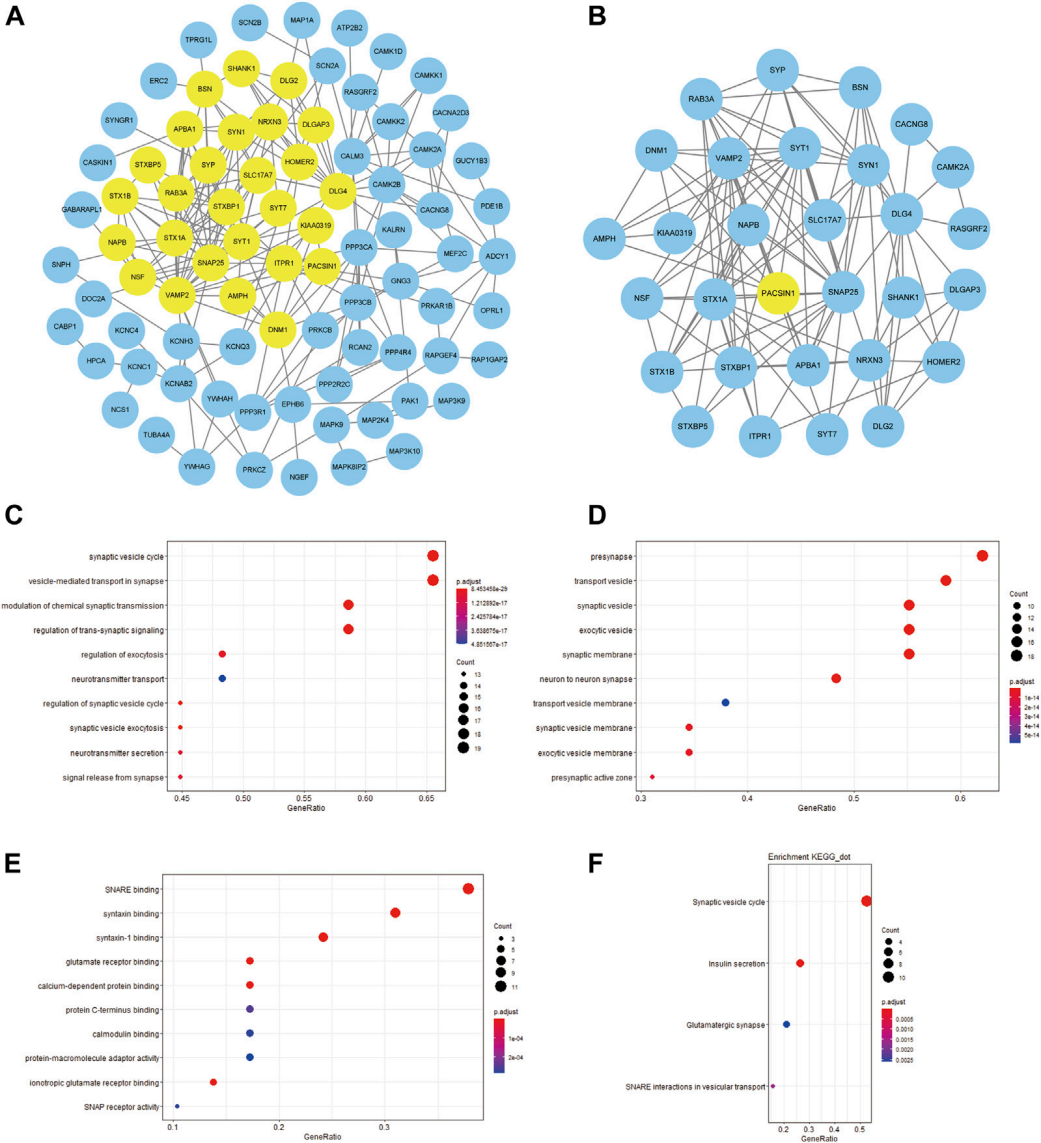
PPI network between PACSIN1 and DEGs and the enrichment analysis of the main gene cluster. The PPI with 84 nodes plus 223 edges was processed with Cytoscape v.3.8.0, and the main gene cluster analysed by MCODE was colored by yellow **(A,B)**. The main gene cluster in GO enrichment analysis results BP **(C)**, CC **(D)**, MF **(E)**, KEGG enrichment analysis results **(F)**.

In order to build a PPI network, we uploaded PACSIN1 and 224 DEGs to the STRING website, and set the interaction score as 0.7. Then, the obtained analysis results were imported into Cytoscape v3.8.0 software to construct an image with 84 nodes plus 223 edges (Figure 8A). We also used MCODE to find out the major gene clusters in the network and finally obtained a total of 30 genes (Figure 8B). Next, we analyzed the functional annotations and signaling pathways of these genes. GO and KEGG enrichment analysis of these genes revealed that they were mainly related to synaptic transmission (Figures 8C,D,E,F), which was consistent with the above research conclusions.

## Discussion

Glioma, the most common subtype of primary brain tumour, is a highly invasive and neurodestructive tumour, and is considered one of the most lethal cancers in humans (Ostrom et al., 2015). According to the Stupp research group results, although surgery, postoperative radiotherapy and chemotherapy can improve the survival rate of glioma patients, the prognosis of most glioma patients is still poor (Stupp et al., 2005). At present, many studies have shown that targeted therapy can improve the efficacy of glioma treatment (Lin et al., 2017). In the DEGs screening of CGGA and GSE4290 datasets between the tumour group and the normal group, we obtained the gene PACSIN1 and found that it significantly downregulated in gliomas (Figures 1A,B). Previously, PACSIN1 was only recognized to affect neurons development and nervous system regulation (Pérez-Otaño et al., 2006; Gopalakrishnan et al., 2016; Koch et al., 2020), and its relationship with tumours or even gliomas has not been explored.

In order to further explore the relationship between PACSIN1 and gliomas, we retrospectively analyzed a total of 1,546 glioma patients from CGGA, TCGA, GSE16011, GSE58218 datasets and 15 glioma samples. First, we investigated the relationship between PACSIN1 and clinical indicators of gliomas (grades, IDH1 mutation status, 1p/19q codeletion status and different molecular subtypes defined by TCGA network (Verhaak et al., 2010)). In 2016 the world health organization (WHO) divided gliomas from low to high into grade I (the lowest degree of malignancy, the best prognosis) to grade IV (the highest degree of malignancy, the worst prognosis). The four grades are based on “genotype molecules” and “histological phenotypes” combination and the “comprehensive” judgment (Louis et al., 2014) according to the histopathological characteristics and malignant degree, including glioblastoma corresponding grade IV group. The results showed that the expression level of PACSIN1 was significantly decreased in different grades (Figures 1C,D,E, Figure 2). Besides, according to IDH mutation status, gliomas can be classified into IDH1 wild-type, IDH1-mutant and NOS (meaning that there is not enough information to define the entity). And according to 1p/19q codeletion status, gliomas can be classified into codel and non-codel group. Many studies indicated that IDH1-mutant gliomas and codel gliomas have a better prognosis (Hu et al., 2017; Chang et al., 2018). In our study, it was revealed that PACSIN1 raised in IDH1-mutant group and reduced in 1p/19q non-codel group (Figure 3). Besides, PACSIN1 also downregulated in the mesenchymal molecular subtype group (Figure 4), suggesting that PACSIN1 would be a biomarker of the mesenchymal molecular subtype gliomas, with a more malignant phenotype and a worse prognosis. Moreover, in order to explore the effect of PACSIN1 on the survival of glioma patients, this study analyzed the PACSIN1 expression in the survival time and status of 325 samples in the CGGA dataset and 603 samples in the TCGA dataset, and found that patients with lower expression of PACSIN1 had significantly shorter OS in all grades of gliomas (Figure 5). Univariate and multivariate analyses verified that PACSIN1 could be an independent prognostic factor (Table 1), which has a certain predictive effect on the occurrence and development of glioma patients.

Finally, in order to explore the mechanism of PACSIN1 inhibiting the occurrence and development of gliomas, total 224 DEGs were screened out through correlation analysis, and functional annotation and pathway analysis were conducted. Then we constructed the PPI network and found the main gene cluster, and we also analyzed these genes with functional annotation and pathway analysis. Finally, it was found that PACSIN1 mainly affects the occurrence and development of gliomas through synaptic transmission (Figures 6–8).

Many studies have proved that synaptic transmission influences the development of gliomas. In particular, in 2019, Nature published three studies simultaneously, revealing a remarkable discovery: the formation of synaptic structures between gliomas and neurons will promote tumour growth. The results of Michelle Monje’s research group show that both AMPA receptor-dependent excitatory post-synaptic current and gap junction targeting mechanisms can significantly slow down the proliferation of gliomas (Venkatesh et al., 2019). Thomas Kuner’s research group also detected the presence of a synaptic component containing the AMPA receptor (Venkataramani et al., 2019). And this is not unique to gliomas. The results of Douglas Hanahan’s research group reveal that breast cancer invasion in the brain may also be mediated by NMDA receptors and glutamate transmitters on excitatory synapses (Zeng et al., 2019). All the above studies have proved that tumour cells in the brain can form excitatory synapses with neurons to promote tumour growth. In this study, we conducted functional annotation and pathway analysis of the DEGs of PACSIN1, and found that PACSIN1 affects the occurrence and development of gliomas mainly through synaptic transmission (Figures 6–8). Meanwhile, it has been shown that PACSIN1 can participate in the transport process by regulating AMPA receptor and NMDA receptor (Anggono et al., 2013; Marco et al., 2013; Widagdo et al., 2016). It still needs our further research and exploration whether PACSIN1 could affect synaptic transmission by modulating AMPA receptors and NMDA receptors, and then influence the occurrence and development of gliomas.

Meanwhile, genes such as CAMK2A, NCS1, NPTX1, RAB3A and SYT1 have been found to affect synaptic transmission. Studies have shown that CAMK2A mutations affect dendritic morphology, lead to synaptic defects, and thus affect behavioural changes associated with ASD (Stephenson et al., 2017). NCS1 binds to the guanine exchange factor Ric8a to regulate the number of synapses and affect the release of neurotransmitters (Romero-Pozuelo et al., 2014). NPTX1 can coordinate the increase of synaptic strength and then regulate neuronal activity through T-type voltage-gated calcium channel and two transcription factors SRF and ELK1 (Schaukowitch et al., 2017). RAB3A deficiency can regulate epilepsy and synaptic activity in hippocampal CA1 region by impair excitatory glutamate synaptic transmission and synaptophysin II synergism (Feliciano et al., 2013). SYT1 can regulate C2a and C2b in C2 region, thus affecting targeted synaptic vesicles (Courtney et al., 2019). And to our surprise, studies have proved that these genes have a certain relationship with gliomas and could influence the occurrence and development of gliomas to a certain extent (Kim et al., 2014; Yukinaga et al., 2014; Zhang et al., 2018; Zhou et al., 2019; Li et al., 2021). These studies lead us to wonder if these genes could influence glioma development through synapses, as these findings suggest. Then we analyzed the expression of these genes and PACSIN1 in the CGGA dataset. Unexpectedly, these five genes are the members of the DEGs of PACSIN1, which are positively correlated with PACSIN1 (Figure 6). It makes us curious whether these genes play a certain regulatory role in the influence of PACSIN1 on the occurrence and development of gliomas, which is also our direction for further exploration and verification in the future.

In this study, PACSIN1 was combined with gliomas for the first time. Through the correlation study between gene expression changes and clinical indicators, it was found that PACSIN1 can be used as an independent prognostic molecule of gliomas, and can predict the prognosis and development of patients’ disease to a certain extent, which will be a potential new site for targeted therapy of gliomas. The shortcoming of this study is that we only conducted a preliminary analysis and discussion at the glioma level, but did not deeply explore the tumour types caused by the differentiation of gliomas in the cell morphology. In addition, we did not carry out relevant animal and cell experiments, but only collected human tissue samples for data analysis. The above studies on the correlation between PACSIN1 and gliomas and the relevant mechanism still need to be further investigated in clinical samples. We will further explore the function of this gene, focusing on its role in other cancer and neurological diseases.

This study provides a basis for the following clinical studies on PACSIN1 in gliomas, and offers new ideas for targeted therapy of gliomas. If the PACSIN1 expression can be interfered in the body, so as to regulate the synaptic transmission pathway, it will make an outstanding contribution to the inhibition of the occurrence and development of gliomas, which is of great significance for glioma patients.

## Data Availability

The datasets presented in this study can be found in online repositories. The names of the repository/repositories and accession number(s) can be found in the article/Supplementary Material.

## Abbreviations

AUC, area under the curve; CGGA, Chinese Glioma Genome Atlas; DEGs, differently expressed genes; GEO, Gene Expression Omnibus; GO, Gene Ontology; HR, Hazard ratio; IDH1, Isocitrate dehydrogenase 1; KEGG, Kyoto Encyclopedia of Genes and Genomes; MCODE, Molecular Complex Detection; OS, overall survival; PACSINI, Protein Kinase C and Casein Kinase Substrate in Neurons 1; PPI, protein-protein interaction; ROC, receiver operating characteristic curve; TCGA, The Cancer Genome Atlas
